# Synergistic algicidal effect and mechanism of two diketopiperazines produced by *Chryseobacterium* sp. strain GLY-1106 on the harmful bloom-forming *Microcystis aeruginosa*

**DOI:** 10.1038/srep14720

**Published:** 2015-10-01

**Authors:** Xingliang Guo, Xianglong Liu, Jianliang Pan, Hong Yang

**Affiliations:** 1 State Key Laboratory of Microbial metabolism, and School of Life Science & Biotechnology, Shanghai Jiao Tong University, Shanghai, 200240, P.R. China

## Abstract

A potent algicidal bacterium isolated from Lake Taihu, *Chryseobacterium* sp. strain GLY-1106, produces two algicidal compounds: 1106-A (cyclo(4-OH-Pro-Leu)) and 1106-B (cyclo(Pro-Leu)). Both diketopiperazines showed strong algicidal activities against *Microcystis aeruginosa*, the dominant bloom-forming cyanobacterium in Lake Taihu. Interestingly, these two algicidal compounds functioned synergistically. Compared with individual treatment, combined treatment with cyclo(4-OH-Pro-Leu) and cyclo(Pro-Leu) significantly enhanced algicidal activity, accelerated the increase in intracellular reactive oxygen species (ROS) levels in *M*. *aeruginosa*, and further decreased the activities of antioxidases, effective quantum yield and maximal electron transport rate of *M*. *aeruginosa*. The results also showed that the algicidal characteristics of cyclo(4-OH-Pro-Leu) are distinct from those of cyclo(Pro-Leu). Cyclo(4-OH-Pro-Leu) mainly interrupted the flux of electron transport in the cyanobacterial photosynthetic system, whereas cyclo(Pro-Leu) mainly inhibited the activity of cyanobacterial intracellular antioxidases. A possible algicidal mechanism for the synergism between cyclo(4-OH-Pro-Leu) and cyclo(Pro-Leu) is proposed, which is in accordance with their distinct algicidal characteristics in individual and combined treatment. These findings suggest that synergism between algicidal compounds might be used as an effective strategy for the future control of *Microcystis* blooms.

Cyanobacterial blooms have become a worldwide aquatic environmental problem due to increasing water eutrophication and climate change^1^,^2^. Lake Taihu, the third largest freshwater lake in China, with a surface area of 2338 km^2^ 3, is no exception. With the discharge of nutrient-rich municipal and industrial wastewater and overapplication of chemical fertilizers around the basin, Lake Taihu has undergone severe eutrophication, leading to frequent large scale outbreaks of cyanobacterial blooms^4^. *Microcystis* is the dominant bloom-forming cyanobacterium in Lake Taihu^5^; *Microcystis* spp. blooms have also attracted global concern due to their adverse influence on ecology and health^4^,^6^.

Nutrient input control is the ultimate strategy for the control of harmful algal blooms (HABs)^7^. Meanwhile, numerous other strategies have been used to manage HABs. Given that the application of chemical (eg. copper sulfate and sodium hypochlorite) and physical methods (eg. clays and flocculants) has been challenged due to the costs and secondary pollution^8^, biomanipulation technologies have received particular attention because of their potential efficacy and eco-friendliness^8^,^9^. Algicidal bacteria are considered to be associated with the regulation and termination of blooms in some lakes^10^,^11^. Recently, many algicidal bacteria have been isolated^9^,^12^,^13^,^14^, and some of the corresponding algicidal substances have also been purified and identified, including pigments^14^,^15^, diketopiperazines^16^,^17^, proteins^18^,^19^, and other complex compounds^9^,^20^.

In our previous work, five algicidal bacterial strains were isolated from Lake Taihu, and four algicidal substances were separated and identified from the algicidal bacteria: pigment (isatin)^14^, diketopiperazines (cyclo(Pro-Gly) and cyclo(Pro-Val))^16^ and hydroquinone^17^. Recently, a potent algicidal strain, *Chryseobacterium* sp. strain GLY-1106, was isolated from Lake Taihu, which produces two algicidal compounds. More interestingly, the combination of these two algicidal substances showed an enhanced algicidal effect, indicating that synergism existed between the two compounds. Accordingly, this study focused on the possible mechanism involved in the observed algicidal synergism of these two algicidal compounds.

## Results

### Isolation and identification of algicidal bacteria

A total of 212 isolates were obtained from surface water samples collected from Meiliang Bay of Lake Taihu in October 2012. Among these strains, approximately 8% (17 isolates) exhibited strong algicidal activity (*A*) (*A* ≥ 90%, *t* = 6 days) against *Microcystis aeruginosa* 9110. The strain GLY-1106, which had the strongest algicidal activity (*A* = 98.9%, *t* = 6 days) (see supplementary Fig. S1a online), was selected for further in-depth investigation. This strain formed shiny, compact yellow colonies with entire edges (see supplementary Fig. S1b online), and the morphological examination (see supplementary Fig. S1c online) via transmission electron microscopy revealed that strain GLY-1106 was a rod-shaped (0.5 to 1.0 by 3.0 to 4.0 μm) bacterium. Bacterial growth occurred under both aerobic and anaerobic conditions, but did not take place at temperatures of ≥37 °C. The Gram-negative isolate hydrolyzed casein and was phosphatase-, catalase- and oxidase-positive, but did not show arginine-, ornithine- and lysine- decarboxylase or phenylalanine deaminase activity. Acid was formed from D-glucose, but no gas was formed. The strain did not produce hydrogen sulfide from triple-sugar iron agar. When the 16S rRNA gene sequence was compared with the sequence available in the GenBank database, the strain GLY-1106 most closely resembled the type strain *Chryseobacterium piscium* LMG 23089^T^ (99% identity, GenBank accession number NR_042410). Most of the physiological properties of strain GLY-1106 were similar to those of *C*. *piscium* (see supplementary Table S1 online). However, some properties were different: in contrast to *C*. *piscium*, the isolate GLY-1106 utilized maltose and D-glucose rather than acetic acid, and it also tested negative for phenylalanine deaminase activity and did not grow in the presence of 5% NaCl. By combining the physiological and biochemical phenotypic characteristics and the results of phylogenetic analysis (see supplementary Fig. S2 online), the newly isolated strain was designated as *Chryseobacterium* sp. strain GLY-1106. The 16S rRNA gene sequence data of strain GLY-1106 have been submitted to the GenBank database under accession number KP342510. This strain has been deposited in the China General Microbiological Collection Center (CGMCC) under accession number CGMCC-9953.

### Algicidal mode of *Chryseobacterium* sp. strain GLY-1106

As shown in Supplementary Fig. S3, the algicidal activity of the strain GLY-1106 culture (*A* = 98.9%, *t* = 6 days) was slightly higher than that of its cell-free filtrate (*A* = 90.7%, *t* = 6 days), and much higher (*P* < 0.01) than that of its re-suspended washed cells (*A* = 37.5%, *t* = 6 days). No significant (*P* > 0.05) difference was observed between the algicidal activities of cell-free filtrate and heat-treated cell-free filtrate. These results indicated that strain GLY-1106 exhibited algicidal activity mainly by producing heat-stable extracellular algicidal compounds to attack cyanobacterial cells indirectly.

### Algicidal range of *Chryseobacterium* sp. strain GLY-1106

The algicidal activity of strain GLY-1106 was evaluated according to its efficiency in inhibiting on the dominant bloom-forming cyanobacterial species in Lake Taihu and other cyanobacterial and algal species (Table 1). Similar to most of the cyanobacterial species tested, especially those isolated from Meiliang Bay in Lake Taihu, strain GLY-1106 exhibited strong algicidal effects. After 6 days of co-culture, strain GLY-1106 showed the highest algicidal activity (*A* = 98.8%) against *M*. *aeruginosa* 9110 among all the cyanobacterial species tested. In addition, the strain GLY-1106 also exhibited an algicidal effect on *Chlamydomonas* sp. BS3 (*A* = 95.2%, *t* = 6 days), a eukaryotic algal strain from Lake Taihu.

### Extraction and purification of the algicidal compounds

The ethyl acetate extract of the cell-free culture filtrate of strain GLY-1106 showed strong algicidal activity against *M*. *aeruginosa* 9110 on cyanobacterial lawns. Following silica gel chromatography of the extract, one fraction which exhibited algicidal activity was obtained from the effluent of the chromatographic column. When the fraction had been collected and applied to semi-preparative high performance liquid chromatography (HPLC), two primary fractions in the effluent from HPLC exhibited algicidal activity on cyanobacterial lawns, i.e. fractions A (retention time (RT): 20.0–21.5 min) and B (RT: 34.8–37.2 min) (see supplementary Fig. S4 online). Fractions A and B were subsequently treated separately by further-purification HPLC. Finally, two purified substances exhibiting algicidal activity, i.e. 1106-A (RT: 58.2–61.0 min, see supplementary Fig. S5 online) and 1106-B (RT: 65.2–74.5 min, see supplementary Fig. S6 online), were obtained from the effluent of further-purification HPLC, respectively.

### Identification of the algicidal compounds

The molecular structures of 1106-A and 1106-B were resolved by high-resolution electrospray ionization mass spectrometry (ESI-MS), electron ionization mass spectrometry (EI-MS) and nuclear magnetic resonance (NMR).

The positive-mode high-resolution ESI-MS spectrum showed the mass charge ratio (m/z) of 1106-A (M+H)^+^ to be 227.1390 (see supplementary Fig. S7 online), which suggested a molecular formula of C_11_H_18_N_2_O_3_. The EI-MS spectrum of 1106-A indicated that ions at 86 and 170 corresponded to molecule (M)-C_6_H_6_NO_3_ and M-C_4_H_8_ ions, respectively (see supplementary Fig. S8 online). Compared with EI-MS spectrum of 1106-A, there was not an EI-MS spectrum of the compound in the GC/MS library with a higher similar index (SI, SI > 800). The NMR data for 1106-A (see supplementary Fig. S9 online) were in agreement with those previously reported for 7-hydroxy-3-isobutyl-hexahydro-pyrrolo[1,2-a]pyrazine-1,4-dione^21^,^22^.

The positive-mode high-resolution ESI-MS spectrum showed the (M+H)^+^ of 1106-B at m/z 211.1441 (see supplementary Fig. S10 online), which suggested a molecular formula of C_11_H_18_N_2_O_2_. The EI-MS spectrum of 1106-B indicated that ions at 70 and 154 corresponded to molecule (M)-C_7_H_10_NO_2_ and M-C_4_H_8_ ions, respectively (see supplementary Fig. S11a online). The EI-MS spectrum of 1106-B was similar (SI > 960) to that of hexahydro-3-(2-methylpropyl)-pyrrolo[1,2-a]pyrazine-1,4-dione (molecular weight (MW): 210, molecular formula: C_11_H_18_N_2_O_2_) in the GC/MS library (see supplementary Fig. S11b online). The NMR data for 1106-B (see supplementary Fig. S12 online) were in agreement with those previously reported for hexahydro-3-(2-methylpropyl)-pyrrolo[1,2-a]pyrazine-1,4-dione^23^.

**Figure 1:**
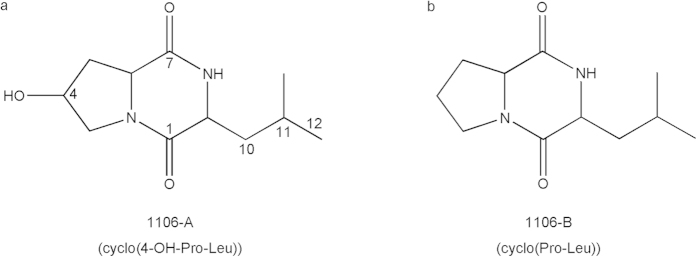
Molecular structures of algicidal compounds produced by *Chryseobacterium* sp. strain GLY-1106.

In summary, 1106-A and 1106-B were identified as 7-hydroxy-3-isobutyl- hexahydro-pyrrolo[1,2-a]pyrazine-1,4-dione (abbreviated as “cyclo(4-OH-Pro-Leu)”) (Fig. 1a) and hexahydro-3-(2-methylpropyl)-pyrrolo[1,2-a]pyrazine-1,4-dione (“cyclo(Pro-Leu)”) (Fig. 1b), respectively.

### Dynamics of the cellular density of strain GLY-1106, concentration of algicidal compounds, and cyanobacterial biomass during the algicidal process

**Figure 2:**
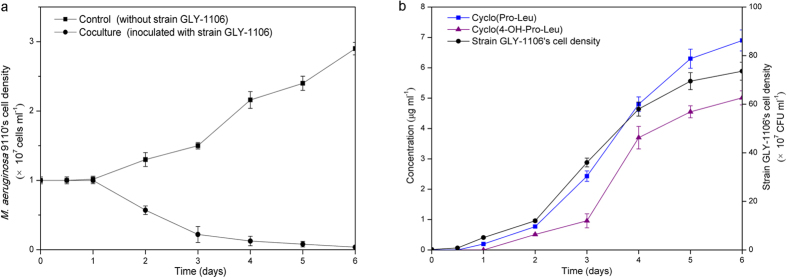
Dynamics of *Microcystis aeruginosa* 9110, strain GLY-1106 and algicidal compounds in the co-culture during algicidal process. (**a**) Growth curve of *M. aeruginosa* 9110 inoculated with algicidal strain GLY-1106 and without strain GLY-1106 (control). Initial concentration of strain GLY-1106 in the cocultures was about 2.0 × 10^6^ CFU ml^–1^. (**b**) Variations of the concentration of algicidal compounds and cell density of strain GLY-1106 in the coculture of *Microcystis aeruginosa* 9110 and strain GLY-1106. The vertical bar represents the standard deviation of triplicate samples.

As shown in Fig. 2, the cellular density of strain GLY-1106 was significantly positively related to the concentration of algicidal compounds (cyclo(4-OH-Pro-Leu): *r* = 0.975, *P* < 0.01; cyclo(Pro-Leu): *r* = 0.994, *P* < 0.01), and significantly negatively related to the cyanobacterial biomass (*r* = −0.948, *P* < 0.01) in general during the algicidal process of strain GLY-1106 against *M*. *aeruginosa* 9110. After 12 h of co-cultivation, when the cellular density of strain GLY-1106 increased to 8.3 × 10^6^ CFU ml^–1^ in the co-culture of *M. aeruginosa* 9110 and strain GLY-1106, the algicidal compounds were still undetectable and the algicidal activities were not observed. After 24 h of co-cultivation, when the cellular density strain GLY-1106 increased to 5.1 × 10^7^ CFU ml^–1^, only the algicidal compounds cyclo(Pro-Leu) was detected, at a concentration of approximately 0.19 μg ml^–1^, leading to weak algicidal activity. When the cellular density of strain GLY-1106 increased to 1.2 × 10^8^ CFU ml^–1^ on the 2nd day, the concentrations of cyclo(4-OH-Pro-Leu) and cyclo(Pro-Leu) reached 0.51 and 0.77 μg ml^–1^, respectively, resulting in a sharp drop in the cellular density of *M*. *aeruginosa* 9110 to 43.8% of the control (without inoculation of strain GLY-1106). After 6 days of co-cultivation, *M*. *aeruginosa* 9110 almost disappeared, the cellular density of strain GLY-1106 increased to 7.36 × 10^8^ CFU ml^–1^ and the concentration of cyclo(4-OH-Pro-Leu) and cyclo(Pro-Leu) rised to 5.03 and 6.92 μg ml^–1^, respectively.

### EC_50_ values of cyclo(4-OH-Pro-Leu) and cyclo(Pro-Leu)

**Figure 3:**
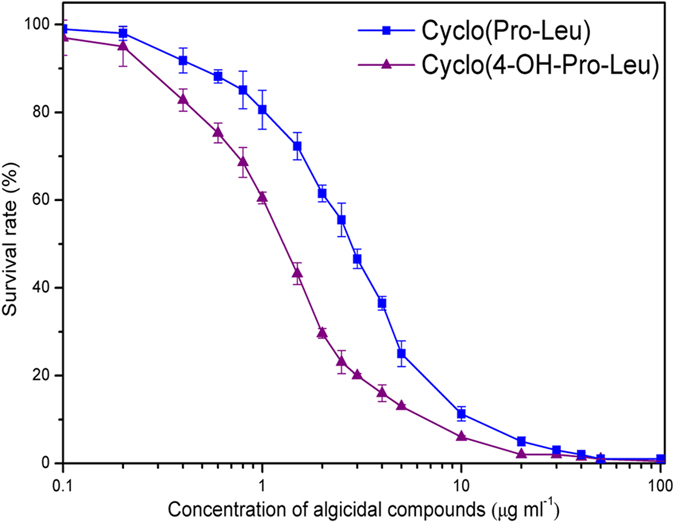
Algicidal effects of cyclo(4-OH-Pro-Leu) and cyclo(Pro-Leu) on the growth of *M. aeruginosa* 9110 after 24 h of exposure.

As shown in Fig. 3, cyclo(4-OH-Pro-Leu) generally showed a stronger algicidal effect than cyclo(Pro-Leu). The EC_50_ values (concentrations for 50% of maximal algicidal effect) of cyclo(4-OH-Pro-Leu) and cyclo(Pro-Leu) calculated from the dose response curve were 1.26 and 2.70 μg ml^–1^, respectively.

### Cyclo(4-OH-Pro-Leu) and cyclo(Pro-Leu) exhibited a synergistic algicidal effect on *M. aeruginosa* 9110

**Figure 4:**
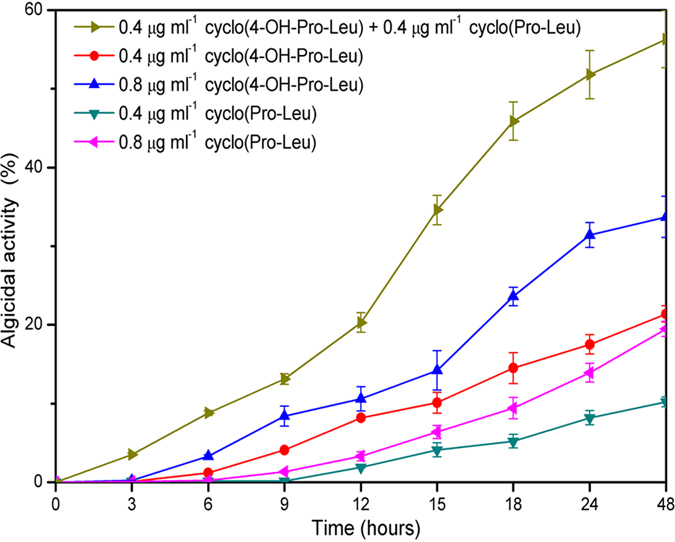
Synergistic algicidal effect of cyclo(4-OH-Pro-Leu) and cyclo(Pro-Leu) on *M. aeruginosa* 9110. All error bars correspond to the standard deviation.

As shown in Fig. 3, individual treatment with cyclo(4-OH-Pro-Leu) and cyclo(Pro-Leu) at a concentration of 0.4 or 0.8 μg ml^–1^ showed a weak or intermediate algicidal activity against *M*. *aeruginosa* 9110. Therefore, the concentration of 0.4 μg ml^–1^ (cyclo(4-OH-Pro-Leu)) plus 0.4 μg ml^–1^ (cyclo(Pro-Leu)) was used in the combined treatment. The results in Fig. 4 indicate that cyclo(4-OH-Pro-Leu) and cyclo(Pro-Leu) exhibited synergistic algicidal effects against *M*. *aeruginosa* 9110. Individual treatment with cyclo(4-OH-Pro-Leu) and cyclo(Pro-Leu) showed obvious algicidal activity after 6 and 9 h of exposure, respectively, while the combined treatment showed obvious algicidal activity after 3 h of exposure. Moreover, the algicidal activity of the combined treatment was significantly (*P* < 0.01) higher than that obtained in the individual treatments at the same time point during the algicidal process. After 48 h of exposure, the algicidal activity of individual treatment with cyclo(4-OH-Pro-Leu) at 0.4 μg ml^–1^ and 0.8 μg ml^–1^ was 21.4% and 33.7%, respectively, and the algicidal activity of individual treatment with cyclo(Pro-Leu) at 0.4 μg ml^–1^ and 0.8 μg ml^–1^ was 10.2% and 19.5%, respectively. However, the algicidal activity of the combined treatment (0.4 μg ml^–1^ cyclo(4-OH-Pro-Leu) plus 0.4 μg ml^–1^ cyclo(Pro-Leu)) reached 56.3% after 48 h, which was 1.67, 2.63, 2.89 and 5.51 times higher than that of the individual treatment with cyclo(4-OH-Pro-Leu) (0.8 μg ml^–1^), cyclo(4-OH-Pro-Leu) (0.4 μg ml^–1^), cyclo(Pro-Leu) (0.8 μg ml^–1^) and cyclo(Pro-Leu) (0.4 μg ml^–1^), respectively, and was 1.78 times higher than the sum of the activity following individual treatment with cyclo(4-OH-Pro-Leu) (0.4 μg ml^–1^) and cyclo(Pro-Leu) (0.4 μg ml^–1^).

### Cyclo(4-OH-Pro-Leu) and cyclo(Pro-Leu) synergistically enhanced the damage to cyanobacterial physiology

To further investigate the underlying mechanism of the synergistic algicidal effects of cyclo(4-OH-Pro-Leu) and cyclo(Pro-Leu) on *M*. *aeruginosa* 9110, the physiological responses of the cyanobacterium to the individual and combined treatments were determined, including the changes in reactive oxygen species (ROS) level, malondialdehyde (MDA) content, antioxidase activity, effective quantum yield (*Φ*_*e*_), and maximal electron transport rate (rETR_max_).

**Figure 5:**
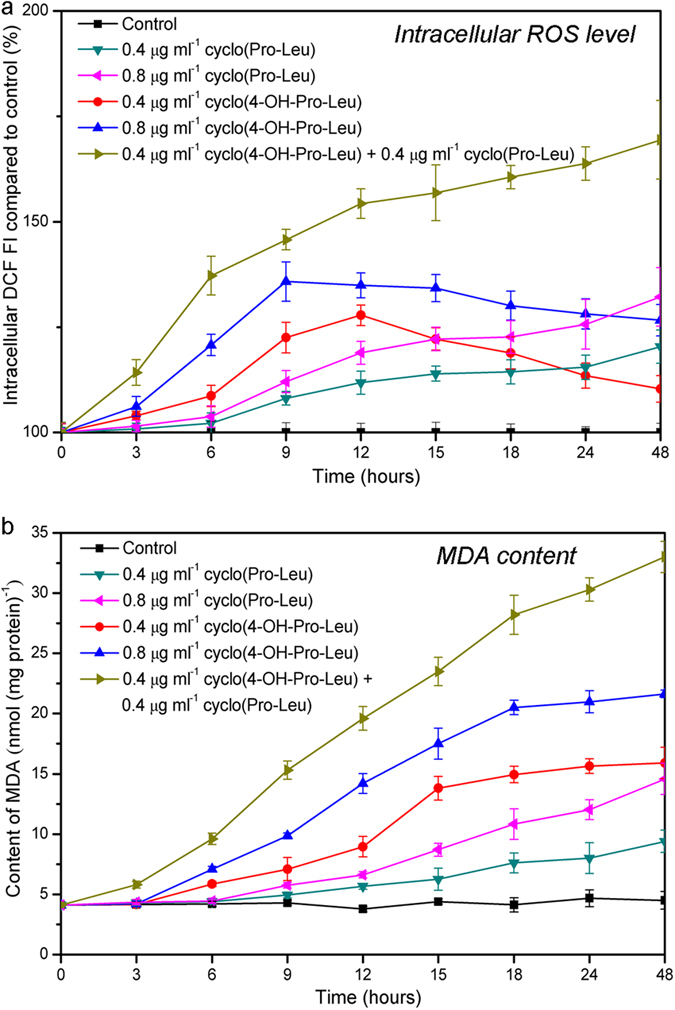
ROS level (a) and MDA content (b) in *M. aeruginosa* 9110 during individual and combined treatments of algicidal compounds. All error bars correspond to the standard deviation.

As shown in Fig. 5a, the intracellular ROS levels in *M*. *aeruginosa* 9110 after individual treatment with cyclo(4-OH-Pro-Leu) at 0.4 μg ml^–1^ and 0.8 μg ml^–1^ reached a maximum after 12 h and 9 h of exposure, respectively, and these two peak ROS levels were 1.28 and 1.36 times higher, respectively, than those of the control at the same time points. The ROS levels after individual treatment with cyclo(Pro-Leu) at 0.4 μg ml^–1^ and 0.8 μg ml^–1^ showed a slowly increase with time. After 48 h of exposure, the ROS levels in *M*. *aeruginosa* 9110 after individual treatment with cyclo(Pro-Leu) (0.4 μg ml^–1^) and cyclo(Pro-Leu) (0.8 μg ml^–1^) were 1.21 and 1.32 times higher than that of the control, respectively. The intracellular ROS level in *M*. *aeruginosa* 9110 with the combined treatment of cyclo(4-OH-Pro-Leu) (0.4 μg ml^–1^) plus cyclo(Pro-Leu) (0.4 μg ml^–1^) exhibited a greater and continuous tendency to increase compared with that after the individual treatments, and the increased ROS levels after the combined treatment were significantly (*P* < 0.01) higher than those after the individual treatments at each time point during the incubation process. After 48 h of exposure, the increased ROS level following the combined treatment was 2.26 times higher than the sum of the individual treatments with cyclo(4-OH-Pro-Leu) (0.4 μg ml^–1^) and cyclo(Pro-Leu) (0.4 μg ml^–1^).

As an indicator of lipid peroxidation^24^, the MDA content in *M*. *aeruginosa* 9110 (Fig. 5b) showed a continuous increase in all the treatments. During the algicidal process, the increased MDA content with the combined treatment (0.4 μg ml^–1^ cyclo(4-OH-Pro-Leu) plus 0.4 μg ml^–1^ cyclo(Pro-Leu)) was significantly (*P* < 0.01) higher than those in the individual treatments. After 48 h of exposure, the increased MDA content after the combined treatment was 1.75 times higher than the sum of the individual treatments with cyclo(4-OH-Pro-Leu) (0.4 μg ml^–1^) and cyclo(Pro-Leu) (0.4 μg ml^–1^).

**Figure 6:**
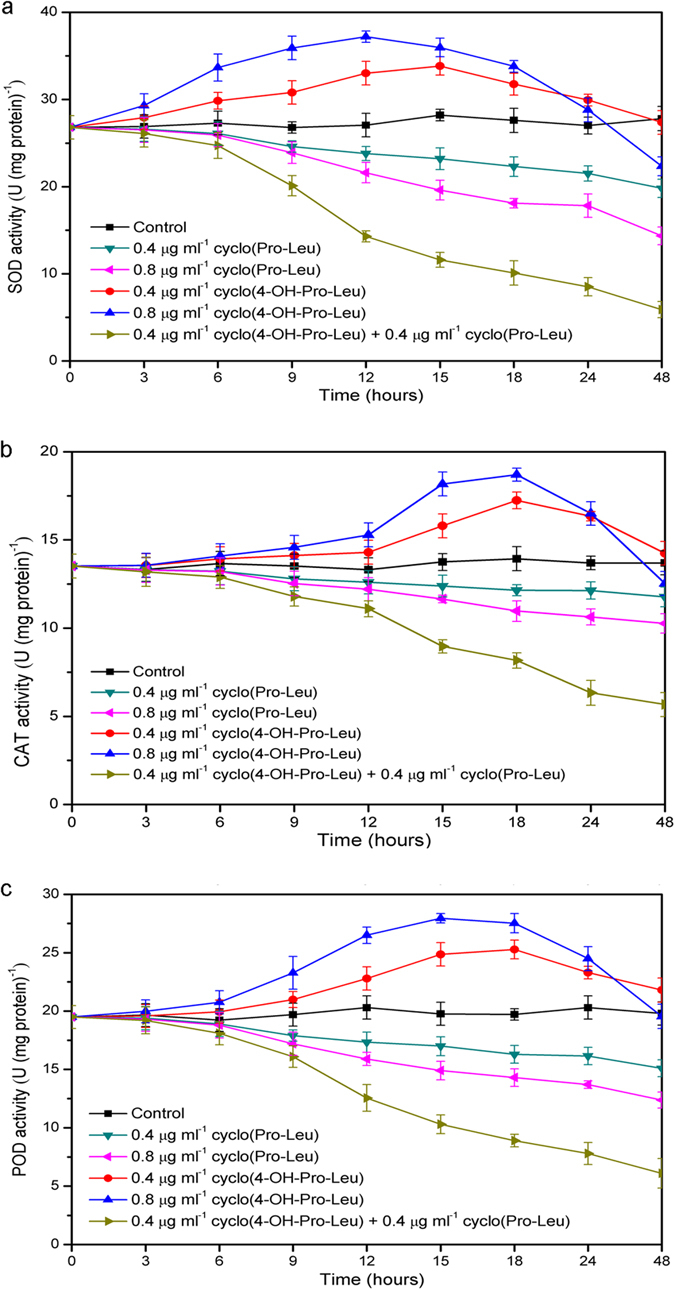
Activities of SOD (a), CAT (b) and POD (c) of *M. aeruginosa* 9110 during individual and combined treatments of algicidal compounds. All error bars correspond to the standard deviation.

As shown in Fig. 6, the response of the antioxidant enzyme system to the algicidal compounds cyclo(4-OH-Pro-Leu) and cyclo(Pro-Leu) was obviously different during the algicidal process. The activities of superoxide dismutase (SOD) (Fig. 6a) following the individual treatment with cyclo(4-OH-Pro-Leu) showed an activating effect compared with the control, and generally exhibited a tendency first to increase then to descend. The SOD activities after the individual treatment with cyclo(4-OH-Pro-Leu) (0.4 μg ml^–1^) and cyclo(4-OH-Pro-Leu) (0.8 μg ml^–1^) reached the highest levels after 15 and 12 h of exposure, respectively. However, the activities of SOD (Fig. 6a) following the individual treatment with cyclo(Pro-Leu) showed an inhibitory effect compared with the control and generally exhibited a tendency to decline throughout the algicidal process. The activities of SOD with the combined treatment (0.4 μg ml^–1^ cyclo(4-OH-Pro-Leu) plus 0.4 μg ml^–1^ cyclo(Pro-Leu)) showed a much greater reducing trends with time. In particular, the activities of SOD after the combined treatment decreased dramatically after 6 h of exposure, and were significantly lower than those following the individual treatments. After 48 h of exposure, the activities of SOD with the combined treatment were only 21.3% of those in the control. During the algicidal process, the activities of catalase (CAT) (Fig. 6b) and peroxidase (POD) (Fig. 6c) varied and resembled those of SOD (Fig. 6a). Following individual treatment with cyclo(4-OH-Pro-Leu), the activities of SOD (cyclo(4-OH-Pro-Leu) (0.4 μg ml^–1^): *r* = 0.722, *P* < 0.01; cyclo(4-OH-Pro-Leu) (0.8 μg ml^–1^): *r* = 0.611, *P* < 0.05) and POD (cyclo(4-OH-Pro-Leu) (0.4 μg ml^–1^): *r* = 0.556, *P* < 0.05; cyclo(4-OH-Pro-Leu) (0.8 μg ml^–1^): *r* = 0.558, *P* < 0.05) were significantly and positively correlated with ROS levels. However, the activities of these three antioxidases were significantly and negatively (*r* = –1.000, *P* < 0.01, in all cases) correlated with ROS levels following individual treatment with cyclo(Pro-Leu) and the combined treatment.

**Figure 7:**
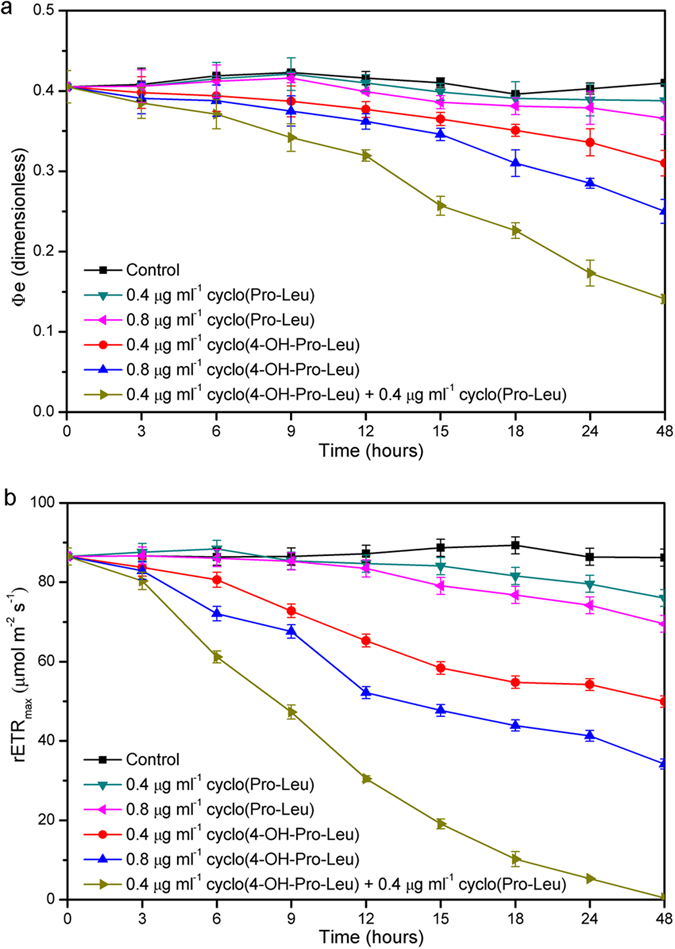
*Φ*_*e*_ (a) (effective quantum yield) and rETR_max_ (b) (maximal electron transport rate) of M. aeruginosa 9110 during individual and combined treatments of algicidal compounds. All error bars correspond to the standard deviation.

As shown in Fig. 7a,b, the *Φ*_*e*_ and rETR_max_ following all treatments showed a tendency to decline during the algicidal process. The *Φ*_*e*_ and rETR_max_ following individual treatment with cyclo(4-OH-Pro-Leu) (0.8 μg ml^–1^) or cyclo(4-OH-Pro-Leu) (0.4 μg ml^–1^) decreased gradually with time, and were 60.9% and 39.7%, or 75.6% and 49.93% of the control after 48 h of exposure, respectively. The *Φ*_*e*_ and rETR_max_ following individual treatment with cyclo(Pro-Leu) (0.8 μg ml^–1^) or cyclo(Pro-Leu) (0.4 μg ml^–1^) began to decrease after 12 h, and were 89.3% and 80.5%, or 94.6% and 88.2% of the control after 48 h of exposure, respectively. The *Φ*_*e*_ and rETR_max_ after the combined treatment (0.4 μg ml^–1^ cyclo(4-OH-Pro-Leu) plus 0.4 μg ml^–1^ cyclo(Pro-Leu)) decreased at a higher rate, and were lower than those following individual treatments at each time point during the algicidal process. In particular, after 6 h, with the highest and continuous increase in ROS level in the combined treatment (Fig. 5a), these two photosynthetic parameters decreased markedly compared with those following individual treatments and the control. After 48 h of exposure, the *Φ*_*e*_ was 34.1% of the control, while rETR_max_ was almost 0% with the combined treatment. During the algicidal process, the decrease in rETR_max_ was greater than that of *Φ*_*e*_ with the combined treatment and individual treatment with cyclo(4-OH-Pro-Leu), but not individual treatment with cyclo(Pro-Leu). In addition, both *Φ*_*e*_ and rETR_max_ were significantly (*r* = −1.000, *P* < 0.01) and negatively correlated with the ROS level following the combined treatment.

## Discussion

Cyclo(4-OH-Pro-Leu) and cyclo(Pro-Leu) are diketopiperazines, which are the simple peptide derivatives. Diketopiperazines constitute a large class of small molecules synthesized by a broad range of microorganisms which show several useful biological properties^25^, and act as antibacterial compounds^26^, antitumor and immunosuppressive agents^27^, and so on. The biosynthesis of diketopiperazines is thought to be catalyzed either by nonribosomal peptide synthetases or by cyclodipeptide synthases^28^. Cyclo(Pro-Gly), produced by *Shewanella* sp. strain Lzh-2^14^ and *Stenotrophomonas* sp. strain F6^17^, and cyclo(Pro-Val), produced by *Bacillus* sp. strain Lzh-5^16^, showed algicidal activity against *M*. *aeruginosa*. Compared with cyclo(Pro-Gly) and cyclo(Pro-Val), cyclo(4-OH-Pro-Leu) and cyclo(Pro-Leu) showed the similar or stronger algicidal activities against *M*. *aeruginosa*. To the best of our knowledge, the cyclo(4-OH-Pro-Leu) and cyclo(Pro-Leu) isolated from *Chryseobacterium* spp. in this study are newly discovered algicidal substances.

Chemicals with similar structures often show distinct biological properties. Xiao and colleagues^6^ isolated a pair of chiral flavonolignans from barley straw, and found that, after short-term exposure, one of the chiral flavonolignans caused significant damage to cyanobacterial cell membranes, but had no influence on the intracellular ROS level, whereas the other chiral flavonolignan induced an increase in intracellular ROS level, but no damage to the cell membrane. In the current study, the difference in the chemical composition between cyclo(4-OH-Pro-Leu) and cyclo(Pro-Leu) is only one hydroxyl group, but their biological properties are vastly different. Synergism may be a common characteristic of some co-occurring secondary metabolites and a powerful driving force in their evolution^29^. Synergism occurring naturally has been observed in bacteriocins, which are peptides of ribosomal origin produced by lactic acid bacteria^30^. The co-produced secondary metabolites of some Actinomycetes act synergistically against either a target microorganism or competitors for nutrients^29^,^31^. In the present study, the combination of cyclo(4-OH-Pro-Leu) and cyclo(Pro-Leu) significantly enhanced the algicidal activity against the bloom-forming *M*. *aeruginosa*. To the best of our knowledge, this is the first report of the synergistic algicidal action of diketopiperazine algicidal substances.

ROS are involved in damage to living organisms under environmental stress^32^. Excessive ROS may cause irreversible oxidative damage to intracellular components, finally leading to cell death^33^. The reaction centers of the photosystem are the major generation sites of ROS in cells^34^. During the photosynthesis process in cyanobacteria, chlorophyll a in photosystem II initially captures photons with sufficient energy (λ < 680 nm), and generates electrons; after this, the electrons are transferred via cytochrom *bf* to photosystem I and are used to produce the NADPH (dihydronicotinamide adenine dinucleotide phosphate)^1^. The photoproducton of ROS can be enhanced by decreased capacity of the electron transport flux^34^. On the other hand, superoxide dismutase (SOD), catalase (CAT) and peroxidase (POD) are three important antioxidases that can scavenge the excessive ROS and protect cells from damage caused by oxygen free radicals^20^. During the algicidal process in the present study, individual treatment with cyclo(4-OH-Pro-Leu) caused a much greater decrease in photosynthetic quantum yield and electron transport rate of *M*. *aeruginosa* than individual treatment with cyclo(Pro-Leu), and the degree of reduction in the electron transport rate was larger than that in the photosynthetic quantum yield, indicating that cyclo(4-OH-Pro-Leu) may induce excessive production of ROS in *M. aeruginosa* mainly by interrupting the electron transport flux. In addition, the antioxidase system was activated by individual treatment with cyclo(4-OH-Pro-Leu) during the algicidal process, and the activities of SOD, CAT and POD were much higher than those following the individual treatment with cyclo(Pro-Leu). In contrast to cyclo(4-OH-Pro-Leu), individual treatment with cyclo(Pro-Leu) caused a significant decrease in antioxidase activities during the algicidal process, but showed a weak or even negligible influence on photosynthetic quantum yield and electron transport rate, which suggests that cyclo(Pro-Leu) may induce excessive levels of ROS in *M*. *aeruginosa* mainly by inhibiting the activities of antioxidases. It can be seen that the algicidal characteristics of cyclo(4-OH-Pro-Leu) are distinct from those of cyclo(Pro-Leu).

Compared with individual treatment with cyclo(4-OH-Pro-Leu) and cyclo(Pro-Leu), the combined treatment not only significantly aggravated the antioxidases activities, but also interrupted electron transport flux in the photosystem of *M*. *aeruginosa*, resulting in a synergistic algicidal effect. With regard to the marked decrease in the activities of antioxidases in the combined treatment after 6 h of exposure, the oxidative damages induced by the continuous increase of ROS level could play a key role in this phenomenon. Accordingly, the mechanism underlying the synergistic algicidal effect of cyclo(4-OH-Pro-Leu) (mainly inhibiting photosynthesis) and cyclo(Pro-Leu) (mainly inhibiting antioxidase activity) may be as follows: both the photosynthetic capacity and antioxidase activities in cyanobacterial cells are inhibited by the two substances simultaneously, accelerating the disruption of redox homeostasis and the increase in intracellular ROS level and lipid peroxidation; in addition, the higher level of ROS may further accelerate the decline in antioxidase activities^35^ and photosynthetic capacity^36^,^37^, resulting in a greater increase in ROS and more oxidative damage in cyanobacterial cells.

Based on the results of this study, it could be seen that the synergism really exists between some algicidal compounds produced by algicidal bacterium, although there was no such phenomenon found in the previous studies on the algicidal compounds derived from algicidal bacteria. Therefore, determining whether or not algicidal synergism exists among more known algicidal compounds derived from algicidal bacteria and exploiting the collaborative mechanism would be of great significance in the future. In addition, synergism between algicidal compounds might be used as an effective strategy for the future control of *Microcystis* blooms.

## Methods

### Cyanobacterial and algal strains, and cultivation conditions

*Microcystis aeruginosa* 9110 (maintained as a unialgal axenic culture, Collection No. CGMCC 9118 in the China General Microbiological Culture Collection Center), *Synechococcus* sp. BN60 (CGMCC 9117), *Chlorophyta* sp. B1, *Oscillatoria* sp. BN35 and *Chlamydomonas* sp. BS3 were isolated from Meiliang Bay in Lake Taihu^13^,^14^. *Chroococcus* sp. FACHB-191, *Microcystis aeruginosa* PCC7806 and *Microcystis viridis* FACHB-979 were purchased from the Freshwater Algae Culture Collection at the Institute of Hydrobiology (FACHB-collection), Chinese Academy of Science, China. All the above-mentioned cyanobacterial and algal strains were maintained in sterilized BG11 medium^14^. Throughout the study, all the cyanobacterial and algal cultures were incubated at 25 °C under 40 μmol photons (m^2^·sec)^–1^ and a 12 h: 12 h (light: dark) cycle^13^.

### Chemicals

Standard cyclo(4-OH-Pro-Leu) (7-hydroxy-3-isobutyl-hexahydro-pyrrolo[1,2-a]pyrazine-1,4-dione) and cyclo(Pro-Leu) (hexahydro-3-(2-methylpropyl)-pyrrolo[1,2-a]pyrazine-1,4-dione) (purity ≥ 99%), which were used for the dose response bioassays and the investigation of the synergistic algicidal effect on *M*. *aeruginosa* 9110, were synthesized by G L Biochem Ltd. (Shanghai, China). The chemicals used for the preparation of all culture media and chemical analysis were purchased from Sigma-Aldrich (St. Louis, MO, USA), if not specifically mentioned.

### Isolation and identification of algicidal bacteria, determination of algicidal mode and range of strain GLY-1106

Water samples were collected from the surface water of the Taihu Ecosystem Research Station (31°24′N, 120°13′E) in hypertrophic Meiliang Bay located in the northeast area of Lake Taihu, using a Ruttner Standard Water Sampler, in October 2012. The water samples collected were immediately transferred into sterile bottles and transported to the laboratory in a mini-icebox. Algicidal bacteria were subsequently isolated from the water samples and identified as described in Supplementary Information online. The methods used for determination of the algicidal mode and range of strain GLY-1106 are detailed in Supplementary Information online.

### Calculation of algicidal activity and survival rate

The algicidal activity (*A*, %) and survival rate (*R*_*s*_, %) were calculated on the basis of the following equations^13^,^14^: *A* = (1 − *D*_*t-treatment*_/*D*_*t-control*_) × 100 and *R*_*s*_(%) = *D*_*t-treatment*_/*D*_*t-control*_ × 100, where *D*_*t*-treatment_ and *D*_*t*-control_ are the cell densities of cyanobacteria in the treatment and control, respectively; *t* (day or hour) represents the inoculation time. Cell densities of *M*. *aeruginosa* (cells ml^−1^) were determined using a hemocytometer under a light microscope (BH-2, Olympus, Japan; Magnification:  × 400). For other cyanobacteria, chlorophyll a (chl-a) concentrations (mg l^−1^) representing the equivalent cell densities were quantified spectrophotometrically using the acetone method^38^.

### Separation, purification and identification of bacterial algicidal compounds, and quantitative analysis of algicidal compounds secreted from strain GLY-1106 by UPLC-MS during the algicidal process

The procedures used for separation, purification and identification of algicidal compounds, and for quantitative analysis of algicidal compounds, were as described in our previous work^14^ with slight modifications as described in Supplementary Information online.

### Dose response bioassays of cyclo(4-OH-Pro-Leu) and cyclo(Pro-Leu) against *M. aeruginosa* 9110

The dose response bioassays were performed with an initial cyanobacterial density of 1 × 10^7^ cells ml^−1^ according to the method by Li and colleagues^14^. Stock solutions of standard cyclo(4-OH-Pro-Leu) and cyclo(Pro-Leu) were prepared by dissolving them in sterile distilled water. An aliquot (0.1 ml) of each stock solution (0.01, 0.02, 0.04, 0.06, 0.08, 0.1, 0.15, 0.2, 0.25, 0.3, 0.4, 0.5, 1.0, 2.0, 3.0, 4.0, 5.0, or 10.0 mg ml^−1^) of standard cyclo(4-OH-Pro-Leu) or cyclo(Pro-Leu) was added to a 9.9-ml culture of *M*. *aeruginosa* 9110 to yield a final concentration of 0.1, 0.2, 0.4, 0.6, 0.8, 1.0, 1.5, 2, 2.5, 3, 4, 5, 10, 20, 30, 40, 50, or 100 μg ml^–1^, respectively. In addition, control (without addition of algicidal compounds) was prepared using an equal amount of sterile distilled water instead of the standard algicidal compound solution. After 24 h of incubation under cyanobacterial growth conditions, the corresponding survival rate (*R*_*s*_) of *M*. *aeruginosa* 9110 was examined. The EC_50_ values were calculated from the relevant dose response curves by probit analysis^39^.

### Synergistic effect of cyclo(4-OH-Pro-Leu) and cyclo(Pro-Leu) against *M. aeruginosa* 9110

To examine whether there was a synergistic effect between cyclo(4-OH-Pro-Leu) and cyclo(Pro-Leu), combined treatment and corresponding individual treatments with these two algicidal compounds were conducted with an initial cyanobacterial density of 1 × 10^7^ cells ml^−1^. Briefly, in the combined treatment, 0.5-ml aliquots of stock solutions (0.08 mg ml^−1^) of standard cyclo(4-OH-Pro-Leu) and cyclo(Pro-Leu) were added to a 99-ml culture of *M*. *aeruginosa* 9110 (99-ml culture of *M*. *aeruginosa* 9110, 0.5 ml of cyclo(4-OH-Pro-Leu), and 0.5 ml of cyclo(Pro-Leu); total, 100 ml), respectively. In the individual treatments, an aliquot (1 ml) of each stock solution (0.04 or 0.08 mg ml^–1^) of standard cyclo(4-OH-Pro-Leu) or cyclo(Pro-Leu) was added to a 99-ml culture of *M*. *aeruginosa* 9110 to yield a final concentration of 0.4 or 0.8 μg ml^–1^, respectively. In addition, 1-ml sterile distilled water was added to a 99-ml culture of *M*. *aeruginosa* 9110 as the control (without addition of algicidal compounds). The cyanobacterial cultures in the treatment groups and control were incubated for 48 h under cyanobacterial growth conditions. During the incubation period, aliquots from both treatment groups and the control were sampled to determine algicidal activities and other relevant physiological indices at the following time points: 0, 3rd, 6th, 9th, 12th, 15th, 18th, 24th, and 48th hour.

### Flow cytometry-based analyses on the intracellular ROS level

Flow cytometric measurements were carried out using a FACSAria II flow cytometer (BD Biosciences, USA) equipped with Diva software for data acquisition. FlowJo software (Tree Star, USA) was used for data analysis. For each sample of cyanobacterial cells, 20,000 events were collected by flow cytometer. Cyanobacterial intracellular ROS formation was determined at the single-cell level following the method described by Wang and colleagues^40^, and the membrane-permeable dye 2′,7′-dichlorodihydroflurescein diacetate (No. D6883, Sigma) was used as a probe. When the probe molecules enter cells, they may be transformed into 2′,7′-dichlorodihydrofluorescein (H_2_DCF) by intracellular esterase. Once the intracellular ROS generated, H_2_DCF would be converted into highly fluorescent 2′,7′-dichlorofluorescein (DCF). Therefore, we determined the fluorescence intensity (FI) of DCF by flow cytometry to indicate the extent of ROS generation. Approximately 10^6^ cells in the treatment groups and control were harvested by centrifugation at 3,000 × g for 10 min, washed twice with sterile phosphate-buffered saline (PBS) solution (50 mM, pH 7.0) and re-suspended in 1 ml PBS (50 mM, pH 7.0). The cyanobacterial suspensions containing 10 μM H_2_DCF-DA were incubated in the dark at 25 °C for 60 min and then washed twice with sterile fresh PBS solution. The stained cells were then analyzed by flow cytometry. Changes in ROS levels as compared with the control were evaluated using the following formula^40^,^41^:


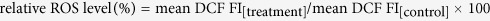


### Determination of antioxidase activity and lipid peroxidation

Cyanobacterial cells were pelleted by centrifugation at 12,000 × g and 4 °C for 20 min and washed twice with PBS (50 mM, pH 7.8). After the pellets had been resuspended in PBS, the cells were disrupted using an Ultrasonic Cell Disruption System (NingBo Scientiz Biotechnological Co., Ltd, China) (200 W, ultrasonic time: 2 s; rest time: 8 s, 30 times) in an ice-bath. The supernatant was then collected by centrifugation for 20 min at 12,000 × g and 4 °C, and used to investigate the physiological changes, including antioxidase activity, total protein and malondialdehyde (MDA, a byproduct of lipid peroxidation) content. Total protein content, concentration of MDA and the activities of superoxide dismutase (SOD), catalase (CAT), and peroxidase (POD) were determined using Total Protein Quantitative assay kit (No. A045-2), Malondialdehyde assay kit (No. A003-1), Total Superoxide Dismutase (T-SOD) assay kit (No. A001-1), Catalase assay kit (No. A007-1), and Peroxidase assay kit (No. A084-3) purchased from Nanjing Jiancheng Institute, China, respectively, according to the manufacturer’s instructions.

### Determination of photosynthetic performance

The photosynthetic performance of photosystem II was determined using a PHYTO-PAM phytoplankton analyzer (Walz, Germany) following the method described by Ou and colleagues^42^. A 5-ml sample of the suspension was analyzed immediately after harvesting. When a sample was acclimated to the light in its environment, effective quantum yield (*Φ*_*e*_) can be calculated as follows:


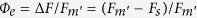


where *F*_*s*_ and *F*_*m*'_ are the corresponding light-acclimated steady-state and maximum fluorescence, respectively, and △*F* is the difference between *F*_*m*'_ and *F*_*s*_. The photosynthetic parameter (*Φ*_*e*_) is an approximation of the fraction of absorbed energy used for photochemistry in the total energy at a specific time and is therefore dimensionless^43^. The other photosynthetic parameter (rETR_max_), which indicates the maximum photosynthetic capacity with the unit of μmol m^–2^ s^–1^ 44, was calculated following the method described by Ralph and Gademann^45^. In practice, these parameters can be obtained directly from the PHYTO-PAM analyzer.

### Data analysis

The data in this study were obtained from three replicates and are presented as means±standard deviation. One-way analysis of variance (ANOVA) was carried out using SAS 9.1.3 (SAS Institute Inc., Cary, NC, USA). Comparisons between the means were conducted using Duncan’s Multiple Range Test. The correlation analysis and probit analysis were conducted using SPSS v 20.0 (IBM Corp., Armonk, NY, USA).

## Additional Information

**How to cite this article**: Guo, X. *et al.* Synergistic algicidal effect and mechanism of two diketopiperazines produced by *Chryseobacterium* sp. strain GLY-1106 on the harmful bloom-forming *Microcystis aeruginosa*. *Sci. Rep.* **5**, 14720; doi: 10.1038/srep14720 (2015).

## Supplementary Material

Supplementary Information

## Figures and Tables

**Table 1:** Algicidal effect of strain GLY-1106 against various cyanobacterial and algal strains.

Stain	Algicidal activity (*A*, %)
*Microcystis aeruginosa* 9110*	98.8 ± 0.6
*Synechococcus* sp. BN60*	87.1 ± 1.3
*Chlorophyta* sp. B1*	97.8 ± 1.7
*Oscillatoria* sp. BN35*	97.6 ± 2.2
*Chlamydomonas* sp. BS3*	95.2 ± 4.1
*Chroococcus* sp. FACHB-191	40.5 ± 2.9
*Microcystis viridis* FACHB-979	90.1 ± 6.2
*Microcystis aeruginosa* PCC7806	89.7 ± 2.5

^*^Isolated from Meiliang Bay of Lake Taihu. The algicidal activities were obtained after 6 days of coculture. Data are the mean ± SD from three independent assays.
