# Supplementary material for: Synergistic algicidal effect and mechanism of two diketopiperazines produced by Chryseobacterium sp. strain GLY-1106 on the harmful bloom-forming Microcystis aeruginosa
Source: Sci Rep. 2015 Oct 1;5:14720. doi: 10.1038/srep14720 (PMC4589682; doi:10.1038/srep14720)
Supplement: Supplementary Information [file srep14720-s1.pdf]

Supplementary Information

Synergistic algicidal effect and mechanism of two diketopiperazines produced by *Chryseobacterium* sp. strain GLY-1106 on the harmful bloom-forming *Microcystis aeruginosa*

Xingliang Guo, Xianglong Liu, Jianliang Pan & Hong Yang*

State Key Laboratory of Microbial metabolism, and School of Life Science & Biotechnology, Shanghai Jiao Tong University, Shanghai, 200240, P.R. China

Correspondence author:

Prof. Dr. Hong Yang

E-mail: hongyang@sjtu.edu.cn (H.Y)

Tel.: (86) 21 34205343

Fax: (86) 21 34205343

Contents:

Supplementary methods Isolation and identification of algicidal bacteria, determination of algicidal mode and range of strain GLY-1106. Separation, purification and identification of bacterial algicidal compounds, and quantitative analysis of algicidal compounds secreted from GLY-1106 by UPLC-MS during the algicidal process.

Table S1. Difference of characteristics between *Chryseobacterium* sp. strain GLY-1106 and *Chryseobacterium piscium* LMG 23089^{T}

Figure S1. (a) The cultures of *Microcystis aeruginosa* 9110 (initial concentration was 1×10^{7} cells ml^{-1}) were grown for 6 days after adding 1% (vol/vol) beef extract peptone medium (control, left flask) and GLY-1106 culture (right flask), respectively. (b) Colony of strain GLY-1106 after 24 h incubation at 28 °C. (c) Transmission electron microscopic observation of the strain GLY-1106.

Figure S2. Neighbour-joining phylogenetic tree based on 16S rRNA gene sequences showing the relationships between strain GLY-1106 (shaded) and closely related species of the genus *Chryseobacterium*. The numbers at the nodes indicate the percentage bootstrap values (above 50%) based on 1000 replicates. GenBank accession numbers are given in parentheses after species name. The tree was rooted with the 16S rRNA gene of *Riemerella columbipharyngis* as an outgroup. The scale bar indicates 0.005 substitution per nucleotide position.

Figure S3. Algicidal effects of differently treated *Chryseobacterium* sp. strain GLY-1106 cultures on *M. aeruginosa* 9110. (A) *M. aeruginosa* cultures with addition

of *Chryseobacterium* sp. strain GLY-1106 cultures; (B) *M. aeruginosa* cultures with addition of *Chryseobacterium* sp. strain GLY-1106 cell-free filtrates; (C) *M. aeruginosa* cultures with addition of *Chryseobacterium* sp. strain GLY-1106 heat-treated cell-free filtrates; (D) *M. aeruginosa* cultures with addition of *Chryseobacterium* sp. strain GLY-1106 cells re-suspended in BG11medium. Data are the mean ± SD from three independent replicates. Mean values denoted by the same letter were not significantly different at $P < 0.05$.

Figure S4. Semi-preparative high performance liquid chromatography (HPLC) of an algicidal fraction from the silica gel column using a Supersil^{TM} C18-EP column (5 μm, 10.0 × 250 mm) and 15% methanol aqueous solution as the mobile phase (a). Algicidal effect of fraction A (retention time (RT) = 20.0−21.5 min), B (RT = 34.8−37.2 min), and control (all other fractions) from the semi-preparative HPLC on a cyanobacterial-lawn, respectively (b). The cyanobacterial-lawns were incubated at 25 °C for 2 days under 40 μmol photons $(m^2 \cdot sec)^{-1}$ and a 12h: 12 h (light: dark) cycle, and RT means retention time, the same as below.

Figure S5. Further-purification HPLC of the fraction A using a Supersil^{TM} C18-EP column (5 μm, 4.6 mm × 250 mm) and methanol-water (5:95 vol/vol) as mobile phase. Arrow indicates the effective peak of secondary active fraction 1106-A (RT = 58.2−61.0 min) on the cyanobacterial-lawn.

Figure S6. Further-purification HPLC of the fraction B using a Supersil^{TM} C18-EP column (5 μm, 4.6 mm × 250 mm) and methanol-water (10:90 vol/vol) as mobile phase. Arrow indicates the effective peak of secondary active fraction 1106-B (RT =

65.2−74.5 min) on the cyanobacterial-lawn.

Figure S7. Positive-mode ESI mass spectrum of 1106-A recorded on LC/MS spectrometers with a ZORBAX Extend-C18 column (Agilent Technologies HPLC 1290-MS 6230, USA).

Figure S8. EI mass spectra of 1106-A by GC/MS analysis.

Figure S9. ^{1}H-NMR (a) and ^{13}C NMR (b) spectra of 1106-A in DMSO-d_6 determined using an NMR spectrometer (400 MHz, Avance Ⅲ, Bruker, Switzerland).

Figure S10. Positive-mode ESI mass spectrum of 1106-B recorded on LC/MS spectrometers with a ZORBAX Extend-C18 column (Agilent Technologies HPLC 1290-MS 6230, USA).

Figure S11. EI mass spectrum of 1106-B by GC/MS analysis (a) and typical spectrum of hexahydro-3-(2-methylpropyl)-pyrrolo[1,2-a]pyrazine-1,4-dione in the GC/MS library (b) whose structure is inserted in the relevant mass spectrum.

Figure S12. ^{1}H NMR (a) and ^{13}C NMR (b) spectra of 1106-B in $CDCl_3$ determined using an NMR spectrometer (400 MHz, Avance Ⅲ, Bruker, Switzerland).

Supplementary methods

Isolation and identification of algicidal bacteria

Water samples were collected from the surface water of the Taihu Ecosystem Research Station (31°24′N, 120°13′E) in hypertrophic Meiliang Bay located at the northeast part of Lake Taihu, using a Ruttner Standard Water Sampler, in October 2012. The collected water samples were immediately transferred into sterile bottles and transported to laboratory in a mini-icebox. Then, algicidal bacteria were screened from the water samples. Briefly, 5 ml of the water sample was inoculated into 95 ml of the exponential-phase culture of *Microcystis aeruginosa* 9110 (1.0×10^7 cells ml^{-1}) and incubated under the cyanobacterial growth conditions described above. As for the negative control, the equal volume sterile distilled water was inoculated instead of water sample. When the cell density of *M. aeruginosa* 9110 declined to below 10% of that in control, 1-ml aliquots of the mixed culture were serially diluted with sterile distilled water and spread onto beef extract peptone (BEP) agar plates (Peptone 10 g, NaCl 5 g, yeast extract 3 g and agar 15 g per litre, pH 7.5), followed by incubation for 24 h at 28 °C. Two hundred and twelve individual colonies with distinct morphology were streaked onto BEP agar plates for further purification and then preserved at -75 °C in the BEP medium containing 15 % glycerol.

Each isolated strain was inoculated into a sterile tube containing 8 ml BEP medium and incubated at 28 °C and 220 r.p.m. for 24 h, and 1 ml of the culture was added into 99 ml of the exponential-phase culture of *Microcystis aeruginosa* 9110 (1.0×10^7 cells ml^{-1}). In the negative control, the equal part sterile BEP medium was

inoculated instead of bacterial culture. After 6 days of coculture, 17 of 212 isolated strains showed algicidal activities, and strain GLY-1106 exhibiting the strongest activity was selected for further study. Cell densities of test strains were quantified using the CFU (colony-forming units) method performed on BEP agar plates[1].

The identification of strain GLY-1106 was accomplished by the combination of phylogenetic and physiological tests[2]. Extraction of bacterial chromosomal DNA from strain GLY-1106, PCR amplification and sequencing of 16S ribosomal RNA (rRNA) gene were performed as previously described[3]. The preliminary identification of strain GLY-1106 was based on the analysis of its 16S rRNA gene sequence using RDP classifier software (version 2.2) and the comparison of the sequence with those deposited in the GenBank database (http://www.ncbi.nlm.nih.gov/blast). 16S rRNA gene sequences of the strains nearest to strain GLY-1106 were retrieved from the NCBI database and aligned by ClustalW[4]. A neighbor-joining tree was constructed using MEGA, version 5.0, software[5]. Meanwhile, the morphological and physiological characteristics were further examined to confirm what species or genus GLY-1106 was attributed to. The physiological tests including enzyme activities, utilization of carbon sources, and others, were conducted according to the methods described by Dong and Cai[2].

The cell morphology was observed using a transmission electron microscope (TEM, Tecnai G2 Spirit Biotwin (120 kv) from FEI Company, USA) according to the method described by Jian and colleagues[6] with some modifications. Briefly, strain GLY-1106 was grown on the BEP agar plate and suspended in 0.85% (wt/vol) sterile NaCl

solution. The prepared cellular suspension was added onto a carbon-coated grid (200 mesh) and stained with 0.5% phosphotungstic acid (PTA). Then the air dried grid was examined under the transmission electron microscope (TEM).

Determination of algicidal mode of strain GLY-1106

The algicidal mode was investigated according to the method described by Lin and colleagues[7] with slight modification. Bacterial culture (at 28 °C and 220 r.p.m, for 24 h) was pelleted by centrifugation at 12,000×g and 25 °C for 20 min and the supernatant was filtered through a sterile 0.22 μm polycarbonate filter to obtain the cell-free filtrate. Heat-treated cell-free filtrate was prepared by autoclaving at 121 °C for 20 min. Bacterial cells were harvested by centrifugation (3,000×g and 25 °C, 10 min), washed twice with sterile BG11 medium and re-suspended in an equal amount of sterile BG11 medium. An aliquot (1 ml) of bacterial cultures, cell-free filtrates, heat-treated cell-free filtrates and washed bacterial cells was inoculated into 99 ml exponential-phase *M. aeruginosa* 9110 cultures respectively and incubated at 25 °C under cyanobacterial growth conditions. *M. aeruginosa* 9110 culture (99 ml) inoculated with 1 ml BEP medium acted as a control. The algicidal activities of various treatments were determined after 6 days exposure.

Determination of algicidal range of strain GLY-1106

The algicidal range of strain GLY-1106 was investigated according to the method described by Tian and colleagues[8] with slight modification. Strain GLY-1106 was inoculated into the sterile BEP medium and incubated at 28 °C and 220 r.p.m. for 24 h. Before being inoculated into the tested cyanobacterial or algal cultures to determine

the algicidal activity, bacterial cells were harvested by centrifugation (3,000×g and 25 °C, 10 min), washed twice with sterile BG11 medium and re-suspended in an equal amount of sterile fresh BEP medium. Then, the bacterial suspensions (1 ml) were inoculated into 99 ml the exponential-phase tested cyanobacterial or algal cultures (Table 1) and incubated at 25 °C under cyanobacterial growth conditions. Initial concentration of GLY-1106 in the cocultures was about 2.0×10^{6} CFU ml^{-1}. Meanwhile, the tested cyanobacterial or algal cultures (99 ml) inoculated with 1 ml BEP medium acted as the corresponding controls. After 6 days of coculture, the algicidal activities were determined.

Separation and purification of bacterial algicidal compounds

The procedure of separation and purification of algicidal compounds was as described in our previous work[3] with slight modifications. During the process of every chromatographic separation, the algicidal peak was found by evaluating the algicidal activity of each effluent fraction which was collected according to the peak shape. If no good peak shape existed at an effluent time, the eluent was collected every half an hour for silica-gel column chromatography or two minutes for high performance liquid chromatography (HPLC). During the whole separation process of algicidal compounds, all effluent fractions in each step were pooled and evaporated, and the algicidal activities of residues' water solution were examined using the cyanobacterial-lawn method[3,8]. The cyanobacterial lawn was developed using the methods described by Li and colleagues[3], and incubated under the cyanobacterial growth conditions.

(ⅰ) *Bacterial culture and ethyl acetate extraction* Strain GLY-1106's culture incubated in BEP liquid medium at 28 °C and 220 r.p.m. for 24 hours was centrifuged at 12,000×g for 20 min to collect the supernatant. The supernatant was extracted three times with an equal volume of ethyl acetate. Then the organic phase was separated, and dried under reduced pressure in a rotary evaporator at 30 °C. After the solvent completely evaporated, the residue was dissolved in the distilled water and filtered through a 0.22 μm membrane filter.

(ⅱ) *Column chromatography* The above filtrate from membrane filter was fractionated by silica-gel column chromatography (commercial silica gel, Qingdao Haiyang Chemical Group Co., 200-300 mesh; 1×50 cm) using an eluent of methanol/chloroform (50:50, vol/vol) at a flow rate of 1 ml min^{-1} and monitored at 254 nm. Algicidal activity of each effluent fraction was evaluated by the cyanobacterial-lawn method.

(ⅲ) *Semi-preparative high performance liquid chromatography (HPLC)* The collected effluent fraction with algicidal activity from silica-gel column was then loaded onto semi-preparative HPLC system (1260 Infinity, Agilent, USA) with a C18 reverse-phase column (Supersil™ C18-EP, 5 μm, 10.0 × 250 mm, Dikma, China), eluted using 15% methanol-water (15:85 vol/vol) at a flow rate of 4 ml min^{-1}, and monitored at 210 nm by the UV-Vis detector (G1314F, Agilent, USA). Algicidal activity of each effluent fraction was evaluated by the cyanobacterial-lawn method.

(ⅳ) *Further-purification HPLC* The algicidal fractions from semi-preparative HPLC were further purified by the further-purification HPLC column (Supersil™

C18-EP, 5 μm, 4.6 mm×250 mm, Dikma, China; flow rate: 1 ml min^{-1}; UV detection at 210 nm). After several rounds further-purification HPLC, the purified algicidal compounds were collected for further structure analysis.

Identification of algicidal compounds

The chemical structures of algicidal compounds were unraveled by combining the high-resolution electrospray ionization mass spectrum (HR-ESI-MS), electron ionization mass spectrum (EI-MS) and nuclear magnetic resonance (NMR).

(ⅰ) *HR-ESI-MS analysis* The purified algicidal compounds were dissolved in methanol for the high-resolution electrospray ionization mass spectrum (HR-ESI-MS) analysis. HR-ESI-MS experiment was performed on ultra-performance liquid chromatography coupled with time-of-flight (UPLC-TOF) mass spectrometer (Agilent Technologies HPLC 1290-MS 6230, USA). Chromatographic separation was performed with ZORBAX Extend-C18 column (1.8 μm, 2.1 × 50 mm) using 10% methanol-water (10:90 vol/vol) as the mobile phase at a flow rate of 0.2 ml min^{-1}, and monitored at 210 nm by the UV-Vis detector. The time-of-flight (TOF) mass spectrometer equipped with ESI source was operated in positive ionization mode (ESI^{+}). The MS data were acquired and processed by the software of MassHunter version 4.0 (Agilent Technologies, USA).

(ii) *EI-MS analysis* Electron ionization mass spectrum (EI-MS) experiment was performed on an Agilent gas chromatography coupled to an electron ionization mass spectrometer (Agilent 6850/5975C) with HP5-MS column (30-m capillary column, 0.25 μm film thickness) and highly pure helium as the carrier gas at a flow of 1 ml

min^{-1}. The ionized samples were analyzed using a mass selective detector after separation by the column. Samples (1 μl) in CH_3OH were injected into the GC-MS. The temperature program was as follows: initially, 50 °C held for 2 min, increased to 100 °C at 10 °C min^{-1} and held for 5 min, and then increased to 300 °C at 10 °C min^{-1} and held for 5 min. Spectra were interpreted by comparison with NIST/EPA/NIH Mass Spec. Library (Version 2.0).

(iii) *NMR analysis* ^{1}H and ^{13}C NMR spectra were recorded on a NMR spectrometer (Avance Ⅲ, Bruker, Switzerland) with setting of 400 MHz for ^{1}H-NMR and 101 MHz for ^{13}C-NMR, using tetramethylsilane as an internal standard at room temperature. The purified algicidal compounds 1106-A and 1106-B were dissolved in DMSO-d_6 and $CDCl_3$, respectively.

Quantitative analysis of algicidal compounds secreted from GLY-1106 by UPLC-MS during the algicidal process

The procedure of quantitative analysis of algicidal compounds was as described in our previous work[3] with certain modifications. Before being inoculated into the culture of cyanobacterium, the cells of strain GLY-1106 was harvested by centrifugation at 3,000×g for 10 min, washed three times with sterile BG11 medium to remove the residual algicidal compounds, and then suspended in the equal amount of sterile fresh BEP medium. Then, 10 ml of GLY-1106's suspension was added into 990 ml the culture of *M. aeruginosa* 9110. Initial concentration of GLY-1106 in the coculture was 2.0×10^{6} CFU ml^{-1}. Meanwhile, 10 ml sterile fresh BEP medium was added into 990 ml of the culture of *M. aeruginosa* 9110 as the control. The cocultures and

control were incubated for 6 days under the cyanobacterial growth conditions. During the period, aliquot portions of both the coculture and control were sampled every day and immediately extracted three times with the equal volume ethyl acetate; then the organic phases were collected and rotary evaporated to full-dryness; after that, the residues were dissolved in 1 ml of distilled water and passed through the 0.22 μm microfiltration membranes to obtain the filtrates. The filtrates and the relevant solutions of standard algicidal compounds were analyzed by ultra performance liquid chromatography coupled with mass spectrometry (UPLC-MS) in positive mode, respectively. The collection and analysis of MS data were performed using the software of MassHunter version 4.0 (Agilent Technologies, USA). The mass chromatograms of algicidal compounds were extracted and integrated from the relevant total ion chromatograms, and the concentrations of algicidal compounds in the filtrates were calculated by comparing their peak areas with those of the standards. The recovery of ethyl acetate extraction of each algicidal compound was evaluated by comparing the peak area of before and after extraction at three levels, i.e., 1.0, 10.0, and 100.0 μg ml^{-1}. The concentration of each algicidal compound in the coculture was calculated on the base of the relevant concentration in the filtrate, the recovery of extraction and the volume of extracted coculture.

References

1. Yamamoto, Y. & Suzuki, K. Distribution and algal-lysing activity of fruiting *myxobacteria* in Lake Suwa. *J. Phycol.* **26**, 457-462 (1990).

2. Dong, X. Z. & Cai, M. Y. *Systematic identification manual of common bacteria*. (Science press, Beijing, 2001).

3. Li, Z. *et al.* A freshwater bacterial strain, *Shewanella* sp. Lzh-2, isolated from Lake Taihu and its two algicidal active substances, hexahydropyrrolo[1,2-a]pyrazine-1,4-dione and 2, 3-indolinedione. *Appl. Microbiol. Biotechnol.* **98**, 4737-4748 (2014).

4. Thompson, J. D., Higgins, D. G. & Gibson, T. J. CLUSTAL W: Improving the sensitivity of progressive multiple sequence alignment through sequence weighting, position-specific gap penalties and weight matrix choice. *Nucleic Acids Res.* **22**, 4673-4680 (1994).

5. Tamura, K. *et al.* MEGA5: molecular evolutionary genetics analysis using maximum likelihood, evolutionary distance, and maximum parsimony methods. *Mol. Biol. Evol.* **28**, 2731-2739 (2011).

6. Jian, H., Xiao, X. & Wang, F. Role of filamentous phage SW1 in regulating the lateral flagella of *Shewanella piezotolerans* strain WP3 at low temperatures. *Applied and environmental microbiology* **79**, 7101-7109 (2013).

7. Lin, S. *et al.* Characterization of an algicidal bacterium *Brevundimonas* J4 and chemical defense of *Synechococcus* sp. BN60 against bacterium J4. *Harmful Algae* **37**, 1-7 (2014).

8. Tian, C. *et al.* Isolation, identification and characterization of an algicidal bacterium from Lake Taihu and preliminary studies on its algicidal compounds. *J. Environ. Sci.* **24**, 1823-1831 (2012).

Table S1. Difference of characteristics between *Chryseobacterium* sp. strain GLY-1106 and *Chryseobacterium piscium* LMG 23089^{T}

Characteristic	*Chryseobacterium* sp. GLY-1106	*Chryseobacterium piscium* LMG 23089^{T} ※
Growth at		
4 °C	w	+
15 °C	+	+
25 °C	+	+
28 °C	+	ND
32 °C	+	+
37 °C	-	-
42 °C	-	-
1% NaCl	+	+
5% NaCl	-	+
pH4.5	-	ND
pH7	+	ND
pH10	-	ND
Enzymes		
Oxidase	+	+
Catalase	+	+
Phosphatase	+	+
Arginine decarboxylase	-	ND
Ornithine decarboxylase	-	ND
Lysine decarboxylase	-	ND
phenylalanine deaminase	-	+
Physiological test		
Casein hydrolysis	+	+
Gelatin hydrolysis	+	+
Glucose acidification	+	ND
Nitrate reduction	-	ND
H_2S production	-	-
Utilization		
D-glucose	w	-
Maltose	+	-
Starch	w	-
Tweens 80	-	-
Trehalose	-	-
Tyrosine	+	-
Cellobiose	w	-
L-arabinose	-	-
Inositol	-	-
Rhamnose	-	-

D-mannose	+	+
Gentiobiose	+	+
Acetic acid	-	+
Sucrose	+	-
D-xylose	-	ND
Sorbitol	-	-
Lactose	w	-
Succinic acid monomethyl ester	+	+

+, positive; -, negative; w, weak; ND, not determined.

※: Data were collected from the following reference:

De Beer, H. *et al. Chryseobacterium piscium* sp. nov., isolated from fish of the South Atlantic Ocean off South Africa. *Int J Syst Evol Microbiol* **56**, 1317-1322 (2006).

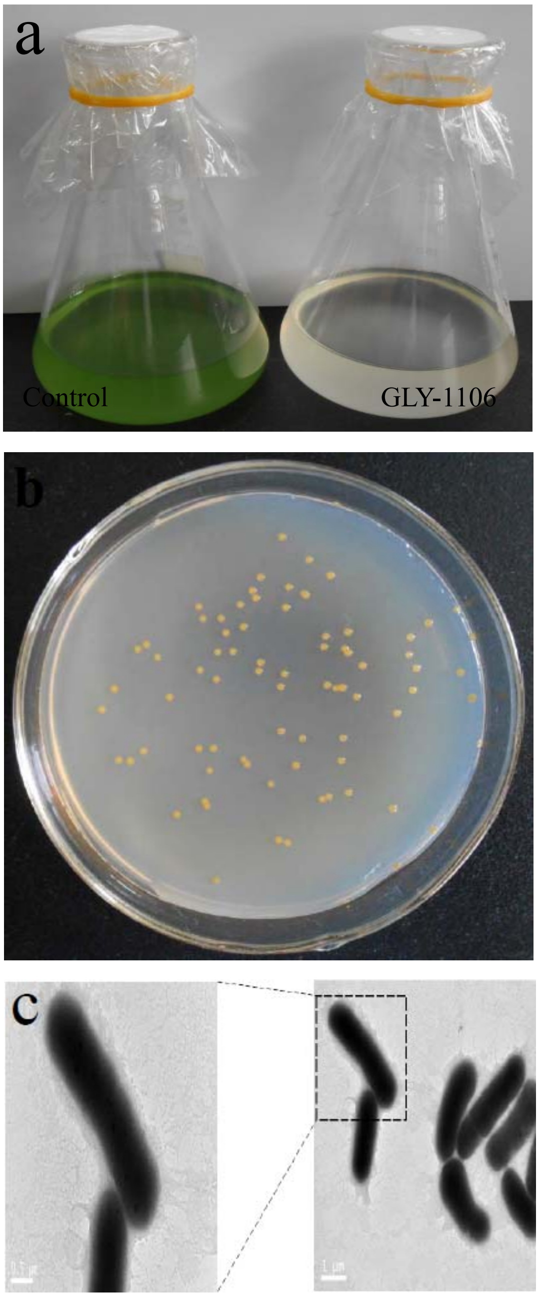

Figure S1. (a) The cultures of *Microcystis aeruginosa* 9110 (initial concentration was 1×10^7 cells ml^{-1}) were grown for 6 days after adding 1% (vol/vol) beef extract peptone medium (control, left flask) and GLY-1106 culture (right flask), respectively. (b) Colony of strain GLY-1106 after 24 h incubation at 28 °C. (c) Transmission electron microscopic observation of the strain GLY-1106.

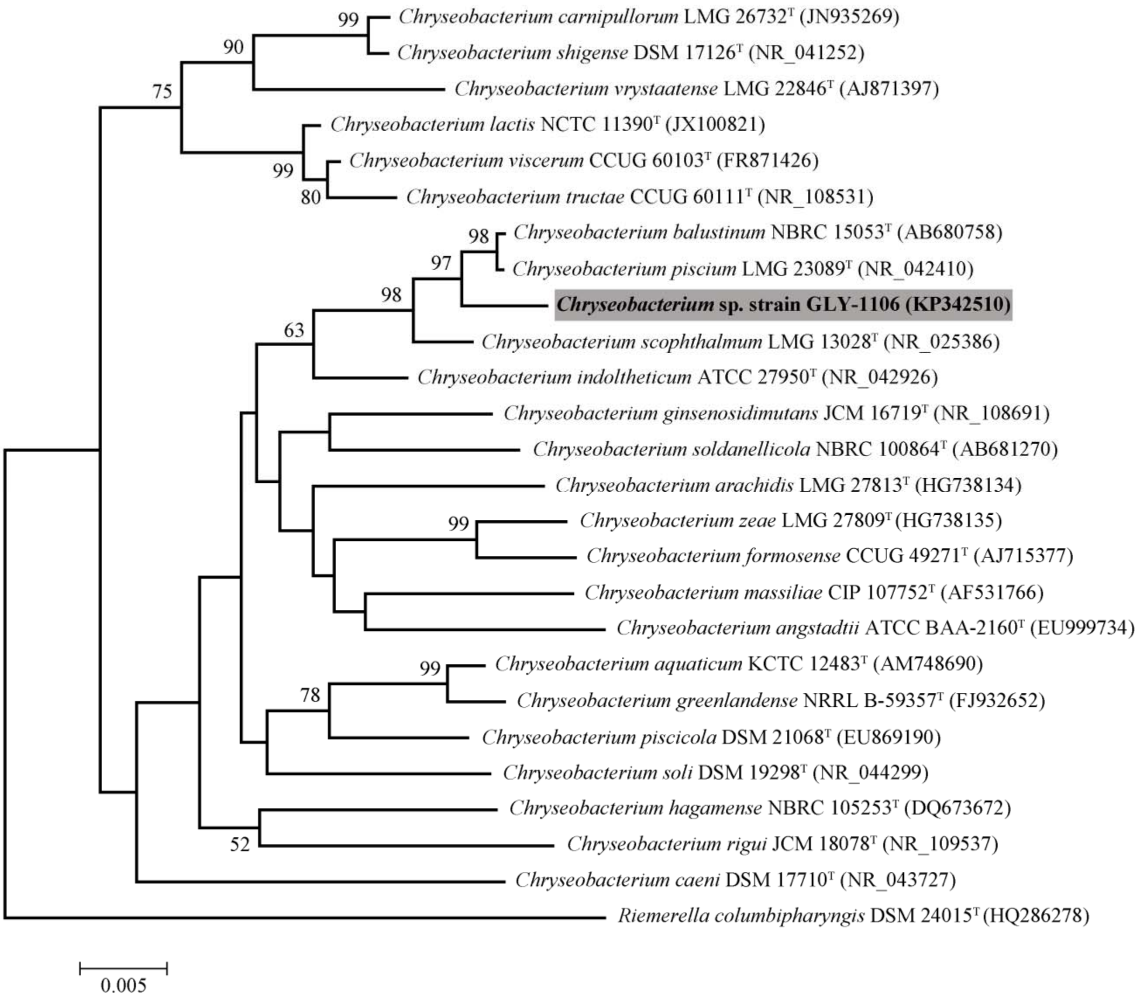

Figure S2. Neighbour-joining phylogenetic tree based on 16S rRNA gene sequences showing the relationships between strain GLY-1106 (shaded) and closely related species of the genus *Chryseobacterium*. The numbers at the nodes indicate the percentage bootstrap values (above 50%) based on 1000 replicates. GenBank accession numbers are given in parentheses after species name. The tree was rooted with the 16S rRNA gene of *Riemerella columbipharyngis* as an outgroup. The scale bar indicates 0.005 substitution per nucleotide position.

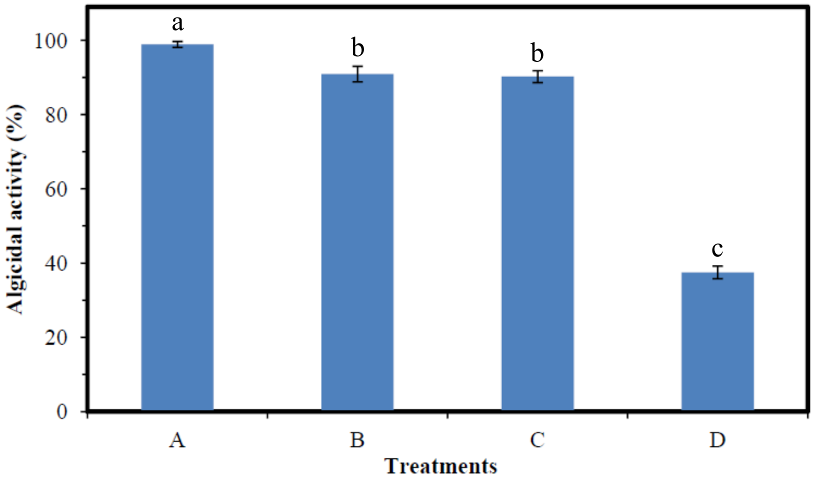

Figure S3. Algicidal effects of differently treated *Chryseobacterium* sp. strain GLY-1106 cultures on *M. aeruginosa* 9110. (A) *M. aeruginosa* cultures with addition of *Chryseobacterium* sp. strain GLY-1106 cultures; (B) *M. aeruginosa* cultures with addition of *Chryseobacterium* sp. strain GLY-1106 cell-free filtrates; (C) *M. aeruginosa* cultures with addition of *Chryseobacterium* sp. strain GLY-1106 heat-treated cell-free filtrates; (D) *M. aeruginosa* cultures with addition of *Chryseobacterium* sp. strain GLY-1106 cells re-suspended in BG11medium. Data are the mean ± SD from three independent replicates. Mean values denoted by the same letter were not significantly different at $P < 0.05$.

(a)

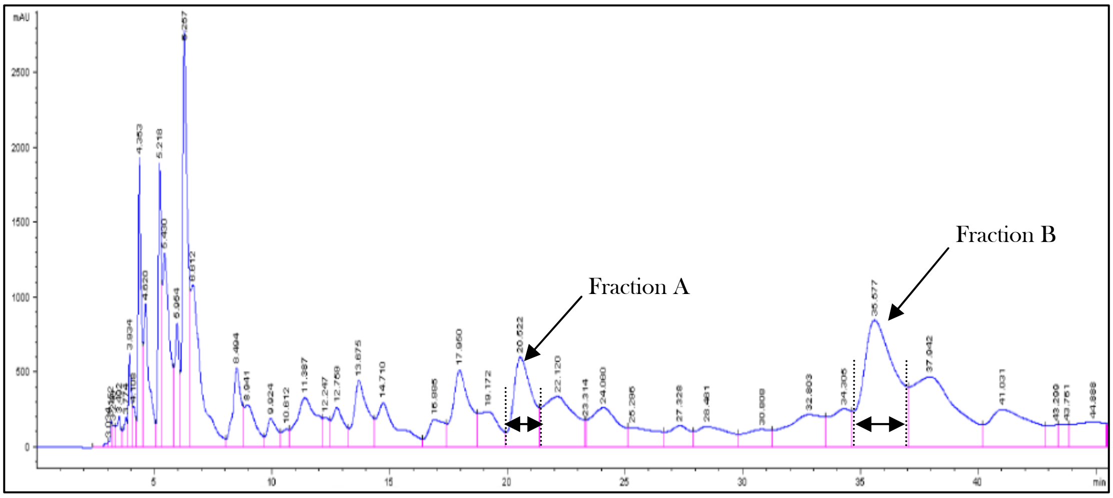

(b)

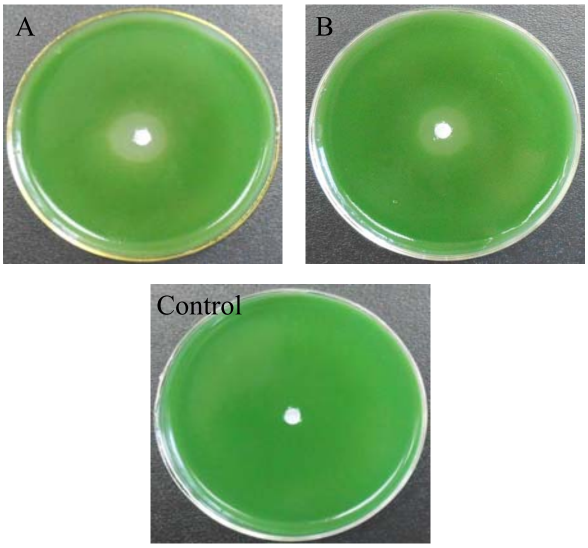

Figure S4. Semi-preparative high performance liquid chromatography (HPLC) of an algicidal fraction from the silica gel column using a Supersil^{TM} C18-EP column (5 µm, 10.0 × 250 mm) and 15% methanol aqueous solution as the mobile phase (a). Algicidal effect of fraction A (retention time (RT) = 20.0–21.5 min), B (RT = 34.8–37.2 min), and control (all other fractions) from the semi-preparative HPLC on a cyanobacterial-lawn, respectively (b). The cyanobacterial-lawns were incubated at 25 °C for 2 days under 40 µmol photons $(m^2 \cdot sec)^{-1}$ and a 12h: 12 h (light: dark) cycle, and RT means retention time, the same as below.

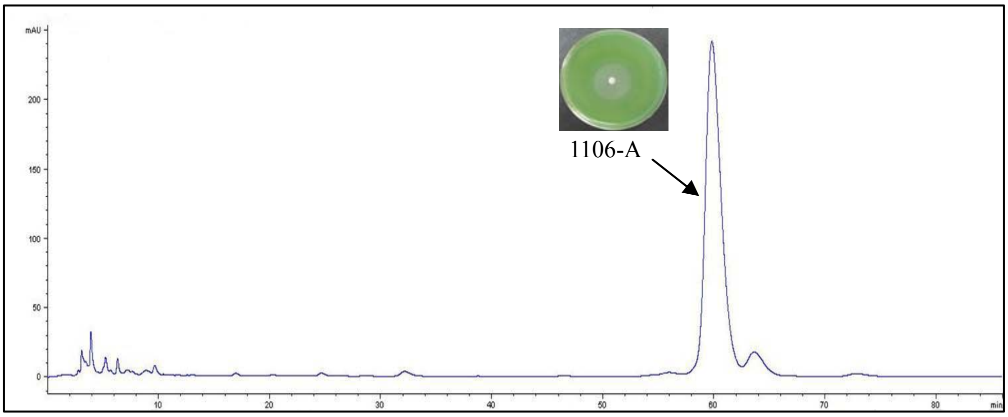

Figure S5. Further-purification HPLC of the fraction A using a Supersil^{TM} C18-EP column (5 μm, 4.6 mm × 250 mm) and methanol-water (5:95 vol/vol) as mobile phase. Arrow indicates the effective peak of secondary active fraction 1106-A (RT = 58.2−61.0 min) on the cyanobacterial-lawn.

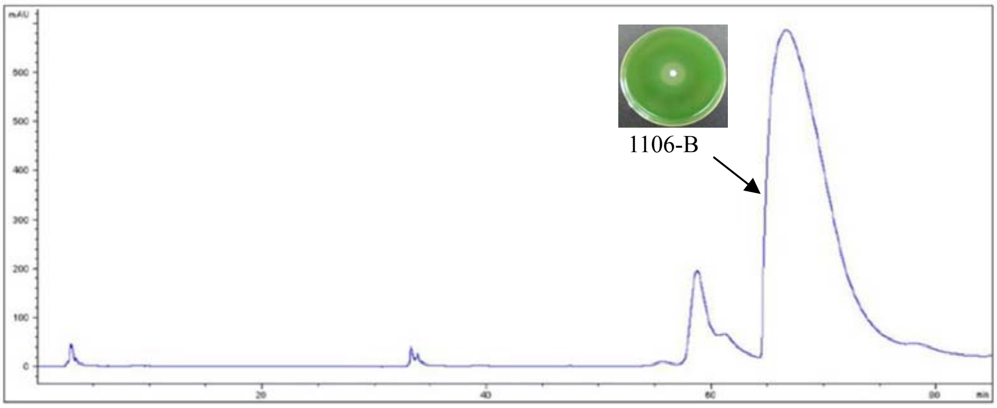

Figure S6. Further-purification HPLC of the fraction B using a Supersil™ C18-EP column (5 μm, 4.6 mm × 250 mm) and methanol-water (10:90 vol/vol) as mobile phase. Arrow indicates the effective peak of secondary active fraction 1106-B (RT = 65.2−74.5 min) on the cyanobacterial-lawn.

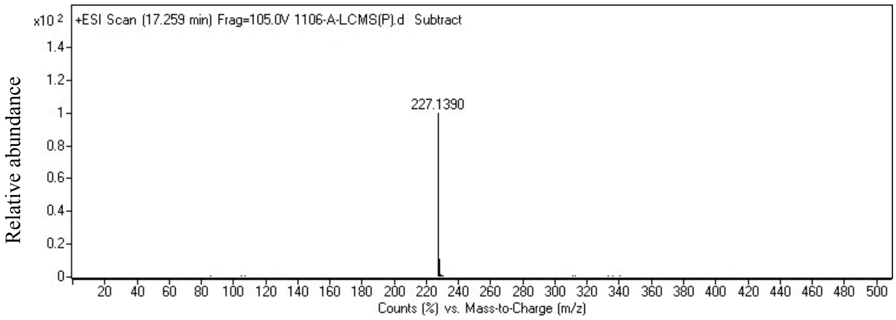

Figure S7. Positive-mode ESI mass spectrum of 1106-A recorded on LC/MS spectrometers with a ZORBAX Extend-C18 column (Agilent Technologies HPLC 1290-MS 6230, USA).

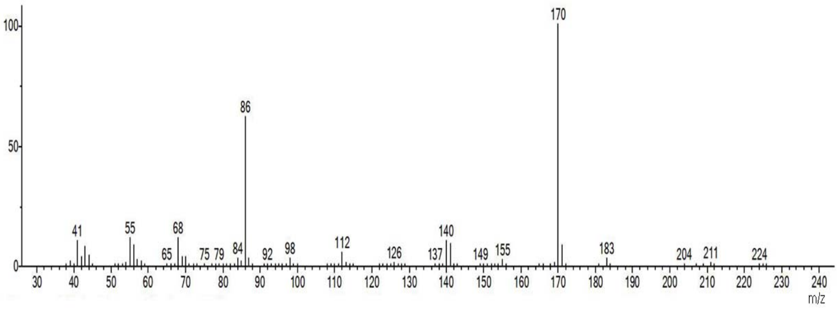

Figure S8. EI mass spectra of 1106-A by GC/MS analysis.

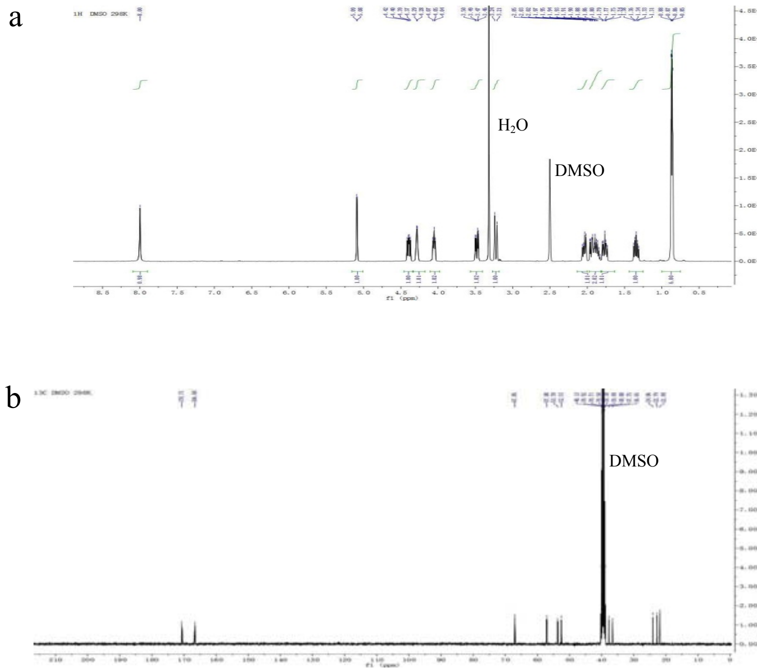

Figure S9. ^{1}H-NMR (a) and ^{13}C NMR (b) spectra of 1106-A in DMSO-d_6 determined using an NMR spectrometer (400 MHz, Avance Ⅲ, Bruker, Switzerland).

NMR data of 1106-A: ^{1}H NMR (400 MHz, dimethylsulphoxide-d_6 (DMSO-d_6)) δ 8.00 (1H, s, N-H), 5.09 (1H, d, J = 2.8 Hz, 4-OH), 4.39 (1H, dd, J = 10.6, 6.7 Hz, H-4), 4.28 (1H, d, J = 2.8 Hz, H-9), 4.05 (1H, t, J = 6.1 Hz, H-6), 3.48 (1H, dd, J = 12.3, 4.4 Hz, H-3a), 3.23 (1H, dd, J = 12.4, 3.0 Hz, H-3b), 2.04 (1H, dd, J = 13.0, 6.7 Hz, H-5a), 1.98-1.82 (2H, m, H-10), 1.77 (1H, ddd, J = 13.5, 8.3, 5.0 Hz, H-11), 1.42-1.25 (1H, m, H-5b), 0.87 (6H, dd, J = 6.5, 2.6 Hz, H-12, H-13); ^{13}C NMR (101 MHz, DMSO-d_6) δ 170.71 (C-1), 166.60 (C-7), 67.05 (C-4), 57.08 (C-6), 53.70 (C-3), 52.53 (C-9), 37.75 (C-10), 36.65 (C-5), 24.06 (C-11), 22.79 (C-13), 21.89 (C-12).

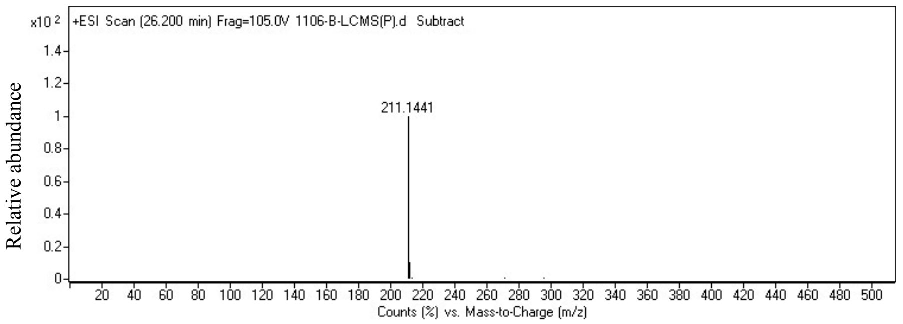

Figure S10. Positive-mode ESI mass spectrum of 1106-B recorded on LC/MS spectrometers with a ZORBAX Extend-C18 column (Agilent Technologies HPLC 1290-MS 6230, USA).

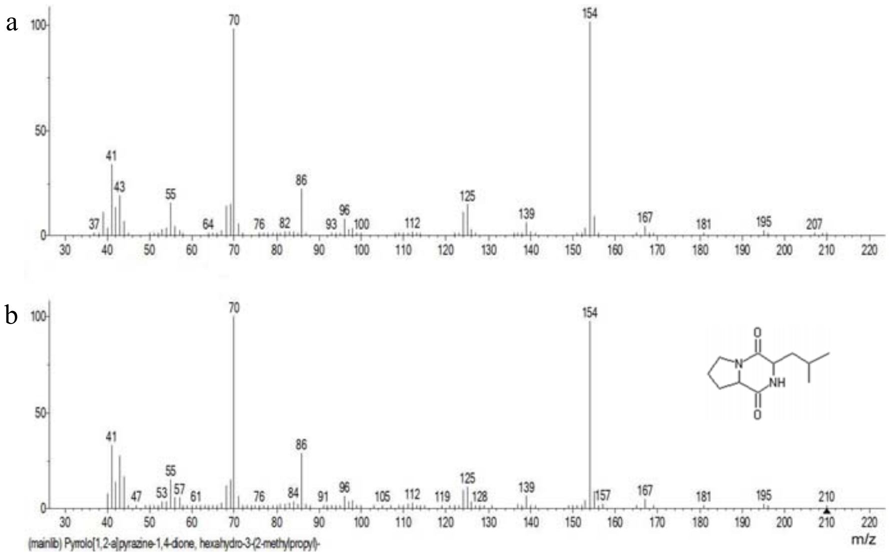

Figure S11. EI mass spectrum of 1106-B by GC/MS analysis (a) and typical spectrum of hexahydro-3-(2-methylpropyl)-pyrrolo[1,2-a]pyrazine-1,4-dione in the GC/MS library (b) whose structure is inserted in the relevant mass spectrum.

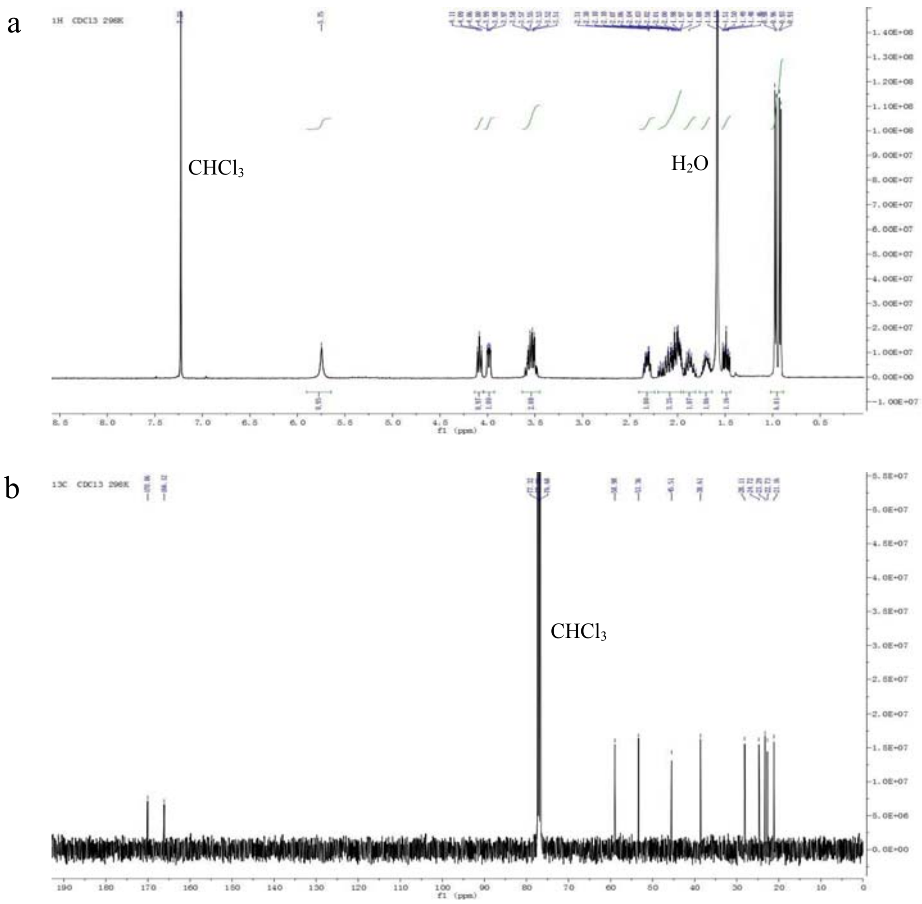

Figure S12. ^{1}H NMR (a) and ^{13}C NMR (b) spectra of 1106-B in $CDCl_3$ determined using an NMR spectrometer (400 MHz, Avance Ⅲ, Bruker, Switzerland).

NMR data of 1106-B: ^{1}H NMR (400 MHz, $CDCl_3$) δ 5.75 (1H, s, N-H), 4.09 (1H, t, *J* = 8.2 Hz, H-6), 3.99 (1H, dd, *J* = 9.5, 3.5 Hz, H-9), 3.67 -3.41 (2H, m, H-3), 2.32 (1H, m, H-5a), 2.22-1.94 (3H, m, H-10a, 5b, 4a), 1.94-1.78 (1H, m, H-4b), 1.77-1.63 (1H, m, H-11), 1.49 (1H, ddd, *J* = 14.5, 9.6, 5.0 Hz, H-10b), 0.97 (3H, d, *J* = 6.6 Hz, H-13), 0.92 (3H, d, *J* = 6.6 Hz, H-12); ^{13}C NMR (101 MHz, $CDCl_3$) δ 170.06 (C-1), 166.12 (C-7), 58.98 (C-6), 53.36 (C-9), 45.51 (C-3), 38.61 (C-10), 28.11 (C-5), 24.72 (C-11), 23.29 (C-4), 22.73 (C-12), 21.16 (C-13).
